# Supplementary material for: Phenotypic manifestation of α-synuclein strains derived from Parkinson’s disease and multiple system atrophy in human dopaminergic neurons
Source: Nat Commun. 2021 Jun 21;12:3817. doi: 10.1038/s41467-021-23682-z (PMC8217249; doi:10.1038/s41467-021-23682-z)
Supplement: Supplementary file 1 — Supplementary Information [file 41467_2021_23682_MOESM1_ESM.pdf]

ID	Diagnosis	*SNCA* genotype	Sex	Biopsy age (years)	Original ID	Reprogramming method	iPSC clone characterisation	GEO
**HC-1**	healthy control	WT/WT	female	67	AH017-7	Cytotune1	Handel et al., 2016 doi:10.1093/hmg/ddv637	GSE69302
**HC-2**	healthy control	WT/WT	female	49	OX3-9	Cytotune1	Dafinca et al., 2016 doi:10.1002/stem.2388	GSE64584
**HC-3**	healthy control	WT/WT	male	67	SFC840-03-03	Cytotune1	Fernandes et al., 2016 doi:10.1016/j.stemcr.2016.01.013	GSE53426
**$SNCA^{A53T}$-1**	PD	A53T/WT	female	51	SFC828-03-09	Cytotune1	Haenseler et al., 2017 doi:10.1016/j.stemcr.2017.05.017	GSE89886
**$SNCA^{A53T}$-2**	PD	A53T/WT	male	46	SFC829-03-06	Cytotune1	Haenseler et al., 2017 doi:10.1016/j.stemcr.2017.05.017	GSE89886
**$SNCA^{TRIP}$-1**	PD	Triplication/ WT	female	55	SFC831-03-01	Cytotune1	Haenseler et al., 2017 doi:10.1016/j.stemcr.2017.05.017	GSE89886
**$SNCA^{TRIP}$-2**	PD	Triplication/ WT	female	55	SFC831-03-03	Cytotune1	Haenseler et al., 2017 doi:10.1016/j.stemcr.2017.05.017	GSE89886
**$SNCA^{TRIP}$-3**	PD	Triplication/ WT	female	55	SFC831-03-05	Cytotune1	Haenseler et al., 2017 doi:10.1016/j.stemcr.2017.05.017	GSE89886
**$SNCA^{TRIP}$-4**	PD	Triplication/ WT	female	55	ND34391G	Cytotune1	Heman-Ackah et al., 2017 doi:10.1093/hmg/ddx331	GSE89886
**$SNCA^{ISOTRIP}$**	PD	Isogenic WT/WT	female	55	Clone 1-13	Cytotune1	Heman-Ackah et al., 2017 doi:10.1093/hmg/ddx331	GSE89886

Supplementary Table 1. iPSC clones used in this study. Each clone was differentiated at least three times.

ID	Diagnosis	Sex	Age at death (years)	Braak staging	Original ID
**PD1**	sporadic PD	male	76	6	NP17
**PD2**	sporadic PD	female	70	2	NP112
**PD3**	sporadic PD	male	80	6	NP133
**MSA1**	MSA-P	male	71	n/a	PD080
**MSA2**	MSA-C	male	64	n/a	PD505
**MSA3**	MSA-P	male	46	n/a	PD515
**MSA4**	MSA-P	male	86	n/a	PD043
**MSA5**	MSA-P	female	67	n/a	PD821

Supplementary Table 2. Post-mortem brain tissue used in this study. Each case was extensively characterised neuropathologically including staining for other proteinopathies. PD cases were sporadic without any SNCA mutations or multiplications.

Description	Forward sequence	Reverse sequence
SNCA	5’-TGTAGGCTCCAAAACCAAGG-3’	5’-GGGGCTCCTTCTTCATTCTT-3’
GAPDH	5’-TGGAAGGACTCATGACCACAG-3’	5’-CAGTGGGGACACGGAAGG-3’
ACTB	5’-CTTCCTGGGCATGGAGTC-3’	5’-GCTCAGGAGGAGCAATGATCT-3’
DJ-1(*PARK7*), sgRNA1 used in HEK cell line	5’-CTGCACAGATGGCGGCTATC-3’	
DJ-1(*PARK7*), sgRNA2 used in HEK cell line	5’-TATGATGTGGTGGTTCTACC-3’	
GLO-1, sgRNA1 used in HEK cell line	5’-TCGTCCGTGAGGCCGCCGGA-3’	
GLO-1, sgRNA2 used in HEK cell line	5’-GCAGACCATGCTACGAGTGA-3’	
non-target control, sgRNA1 used in HEK cell line	5’-GGTATTCCGAAGTCCTCCGC-3’	
non-target control, sgRNA2 used in HEK cell line	5’-GGATACCTGGGCCGACTTTC-3’	
DJ-1 Exon2 for detection assay, DA, Product: 531 bp (WT) or 362 bp (KO) used in iPSC cell line	5’-CCTCATTATGTTTTCATCT CAGAGC-3’	5’-TGGGTCTGAAGGAAATATGGCA-3’
DJ-1 Exon2 for absence assay, AA, product: 464bp (WT) or no band (KO) used in iPSC cell line	5’-CGAGGACTGCCTCAACCAG-3’	5’-CTTGTCTGCCAAGAACCCCT-3’
DJ-1 Exon2 sequencing used in iPSC cell line	5’-TGGGTCTGAAGGAAATATGGCA-3’	
DJ-1 Exon4 for detection assay, DA, Product: 431 bp (WT) or 341 bp (KO) used in iPSC cell line	5’-CAGCGTGGACATCCTT GCAT-3’	5’-CACAGCCTCCTCCCGAAA-3’
DJ-1 Exon4 for absence assay. AA, Product: 284 bp (WT) or no product (KO) used in iPSC cell line	5’-TACTCTGCAGCGTGGACATC-3’	5’-TGTGCGCCCAGATTACCTC-3’
DJ-1 Exon4 sequencing used in iPSC cell line	5’-TGCACAGTTGAAATGAAATG-3’	
DJ-1, sgRNA1 targeting exon2 used in iPSC cell line	5’-TCATCCCTGTAGATGTCATGAGG-3’	
DJ-1, sgRNA2 targeting exon2 used in iPSC cell line	5’-CCACTGCTCACATTTAACGCTTG-3’	
DJ-1, sgRNA1 targeting exon4 used in iPSC cell line	5’-GTTACAGGGACCATATGATGTGG-3’	
DJ-1, sgRNA2 targeting exon4 used in iPSC cell line	5’-CCTCAA TAAAGCTGGGGGGGGGG-3’	

Supplementary Table 3. List of primers and sgRNAs used in this study.

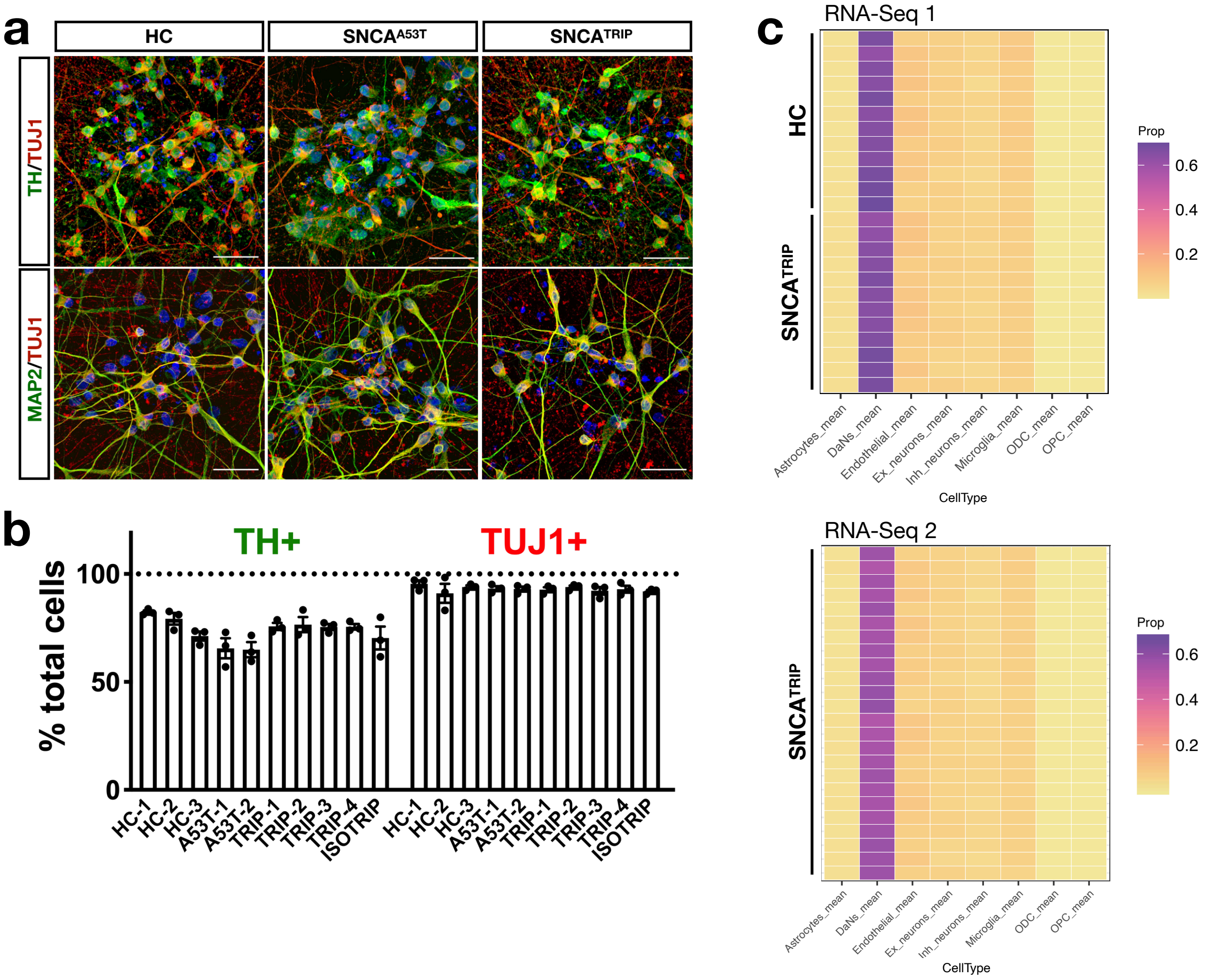

Supplementary Figure 1. Characterisation of iPSC-derived dopaminergic neurons. a, Confocal images of representative differentiated neurons from each line used (healthy control, HC; SNCA^{A53T}; SNCA^{TRIP}) expressed the dopaminergic marker tyrosine hydroxylase (TH) and the neuronal markers microtubule associated protein-2 (MAP2) and beta-3 tubulin (TUJ1). Images are representative of three independent differentiations. Scale bar: 50 μm. **b,** Approximately 76% of neurons from each line were TH-positive and ~93% were TUJ1-positive. Each dot corresponds to one clone differentiated once and data are mean±s.e.m from n=3 differentiations per clone. **c,** Transcriptomic analysis in two bulk RNA-Seq experiments demonstrated that ~70% of neurons were dopaminergic (DaN). Source data for **b** are provided as a Source Data file.

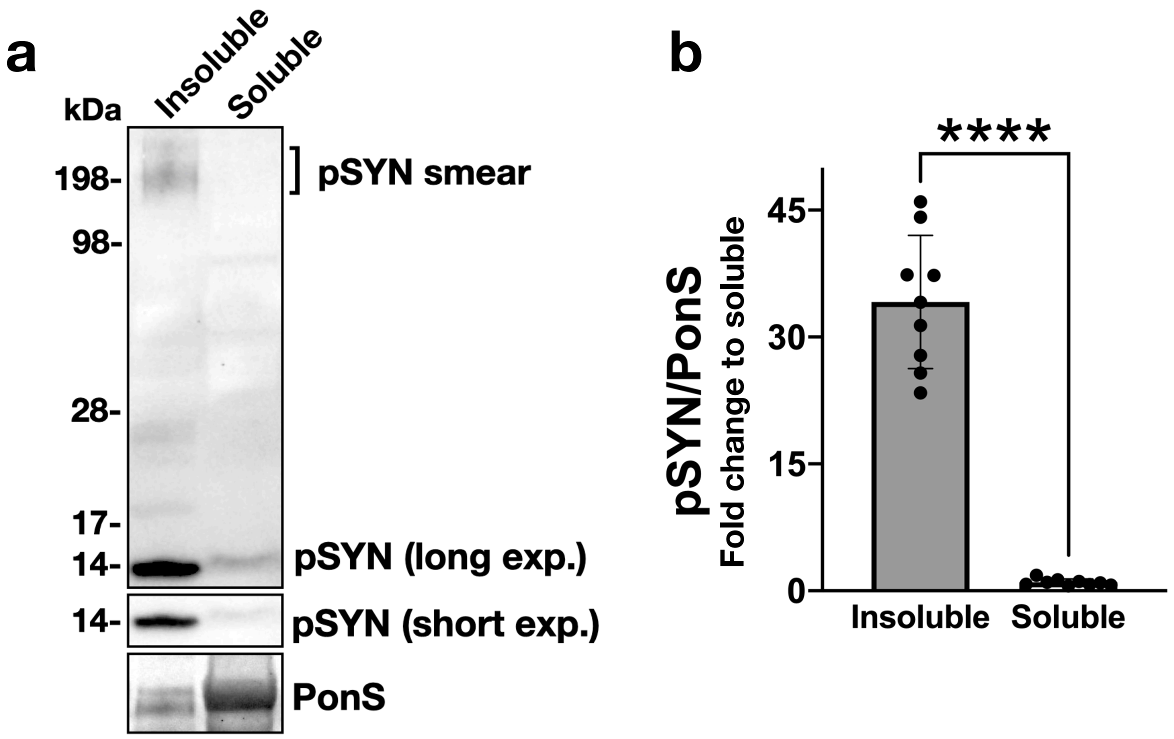

Supplementary Figure 2. Phosphorylated α-synuclein in RIPA-insoluble fraction. a, iPSC-derived dopaminergic neurons from SNCA^TRIP^ were seeded with *de novo* fibrils (1 μM) and lysed in RIPA buffer. Neuronal lysates were fractionated into soluble and insoluble fractions following 100,000g centrifugation. Immunoblotting revealed that p-S129 synuclein (pSYN) was almost exclusively found in the RIPA-insoluble fraction. Also observed in the insoluble fraction was pSYN-positive higher molecular weight smear at 198 kDa. The immunoblot is representative of three independent experiments. **b**, Quantification of immunoblots demonstrated that 97% of pSYN is present in the RIPA-insoluble fraction. Each dot corresponds to one clone differentiated once and data are mean±s.e.m from 3 differentiations per clone. For panel **b**, n=9, ****P<0.0001, two-sided unpaired Student's t-test. Source data for **a** and **b** are provided as a Source Data file.

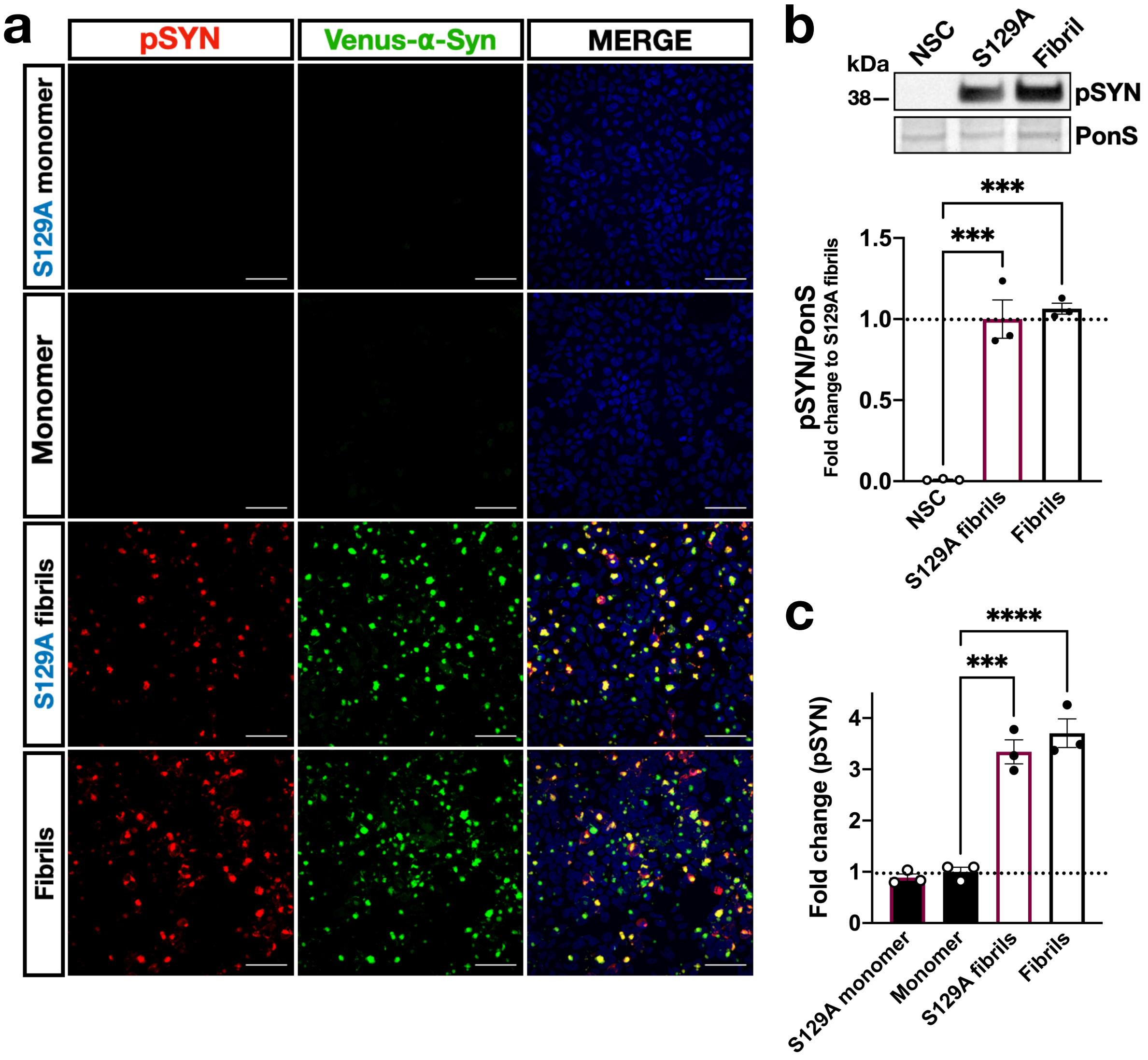

Supplementary Figure 3. S129A mutant *de novo* fibrils induce p-S129 α-synuclein-positive inclusions. a, Confocal images of HEK 293 cells over expressing α-synuclein-Venus treated with S129A mutant human recombinant α-synuclein monomer, wildtype human recombinant α-synuclein monomer, S129A mutant *de novo* fibrils, and wildtype *de novo* fibrils. S129A mutant fibrils seed p-S129 α-synuclein (pSYN) and Venus positive inclusions similarly to wildtype *de novo* fibrils. Images are representative of three independent experiments. Scale bar: 50 *µ*m. **b**, Immunoblotting analysis of HEK 293 cells expressing α-synuclein-Venus demonstrated that S129A mutant and wildtype *de novo* fibrils both seeded aggregates with similar levels of pSYN. Representative immunoblot with quantification from n=3 independent experiments. **c**, iPSC-derived dopaminergic neurons from $SNCA^{TRIP}$ were exposed to either S129A mutant or wildtype monomers or fibrils and analysed through a time resolved fluorescence assay (HTRF) specific to pSYN. S129A mutant fibrils induced similar pSYN levels compared to wildtype *de novo* fibrils. Each dot in panel **c** corresponds to one clone differentiated once (n=3). Data are shown as mean±s.e.m. In panels **b** and **c**, ***P<0.001,****P<0.0001 by one-way ANOVA followed by Tukey's multiple comparison test. Source data for **b** and **c** are provided as a Source Data file.

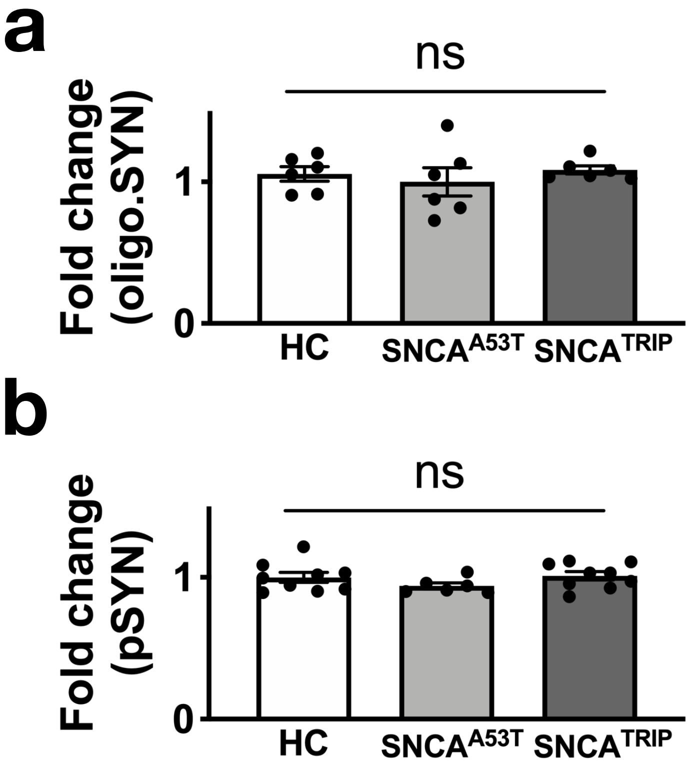

Supplementary Figure 4. No aggregation was detected in iPSC-derived dopaminergic neurons without seeding at DIV45. a, Using time resolved FRET specific to oligomeric α-Synuclein, no aggregation was detected in healthy controls (n=6), $SNCA^{A53T}$ (n=6) and $SNCA^{TRIP}$ (n=6) prior to seeding at DIV45. **b,** No pSYN was detected under the same basal conditions in healthy controls (n=9), $SNCA^{A53T}$ (n=6) and $SNCA^{TRIP}$ (*n*=9). Each dot in panels **a** and **b** corresponds to one clone differentiated once and data are mean±s.e.m from three independent differentiations. ns: non-significant by one-way ANOVA followed by Tukey's multiple comparison test. Data shown as fold-change to healthy control (HC). Source data for **a** and **b** are provided as a Source Data file.

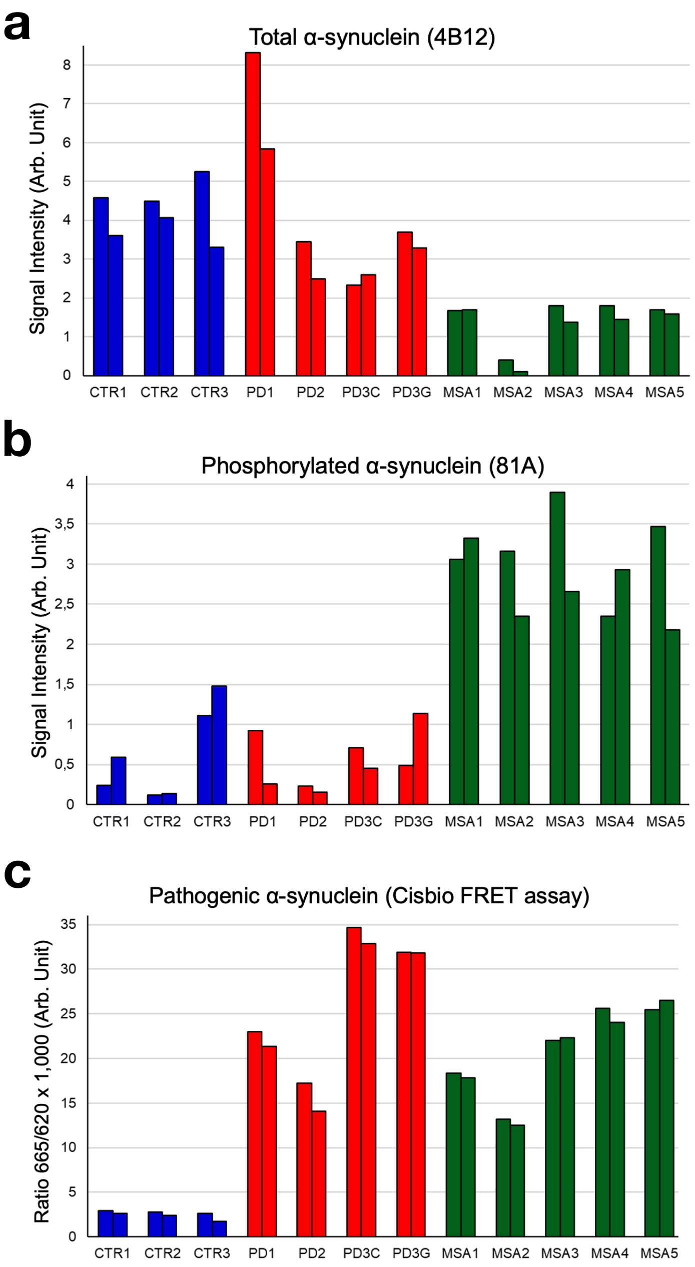

Supplementary Figure 5. Quantification of pathogenic α-synuclein aggregates in patient brains. Brain tissue from 3 PD, 5 MSA (cerebellum) patients as well as 3 age-matched and brain-region matched healthy controls were obtained at autopsy and processed into total brain homogenates. For one PD case both cingulate cortex (C) and temporal gyrus (G) was used. **a** and **b**, The amount of pathogenic, aggregated α-synuclein in the different brain homogenates was determined using a filter retardation assay (n=2). 50 *μ*l of brain homogenates (1% in PBS, weight:volume), equivalent to 0.5 mg of brain homogenate, were filtered on cellulose acetate membranes and probed with **a**, 4B12 (total α-synuclein) or **b**, 81A (phosphorylated α-synuclein) antibodies. **c**, The amount of pathogenic, phosphorylated, α–synuclein in the different brain homogenates (10% in PBS, weight:volume) was also quantified using the Cisbio FRET assay (n=2). Source data for **a, b,** and **c** are provided as a Source Data file.

a

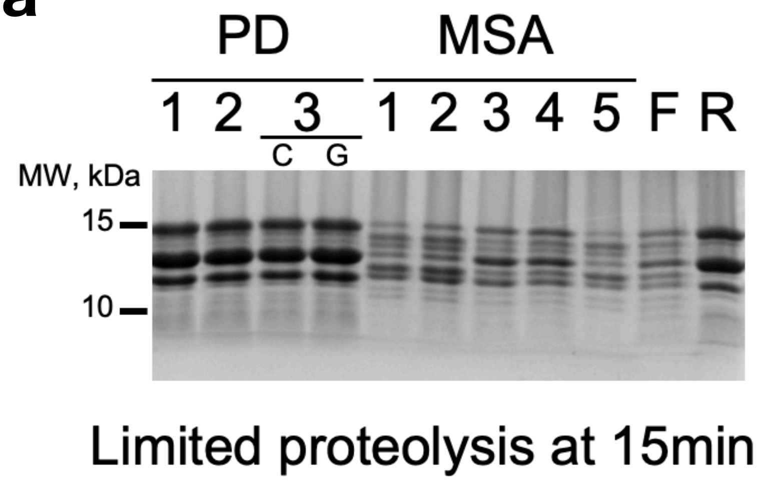

b

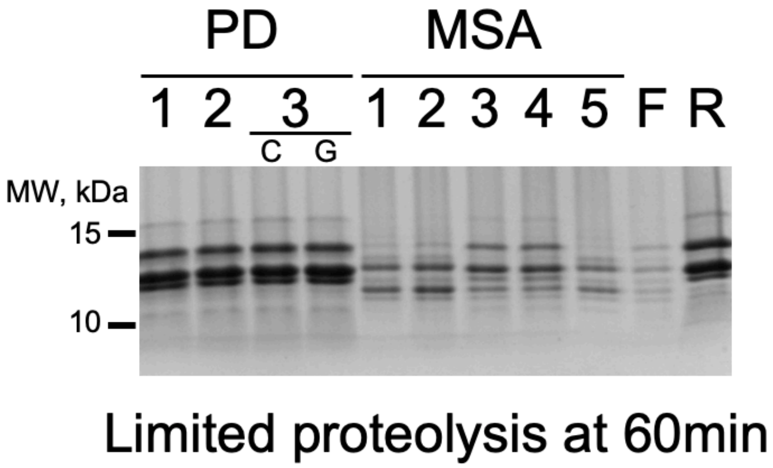

Supplementary Figure 6. Limited proteolysis of PMCA-amplified fibrils. Proteolytic patterns of PMCA-amplified fibrils from PD and MSA brains, fibrils (F) and ribbons (R) after the 3rd cycle. Proteinase K concentration was 3.8 μg/ml. Samples were withdrawn from the reaction, immediately after PK addition. PAGE analysis was performed as described in the methods section and the gels were stained with Coomassie blue. Panels **a** and **b** represent a single blot containing the preparations from each amplification run together (n=4 for PD and n=5 for MSA). Proteolytic patterns are shown at **a**, 15 min and **b**, 60 min. Source data for **a** and **b** are provided as a Source Data file.

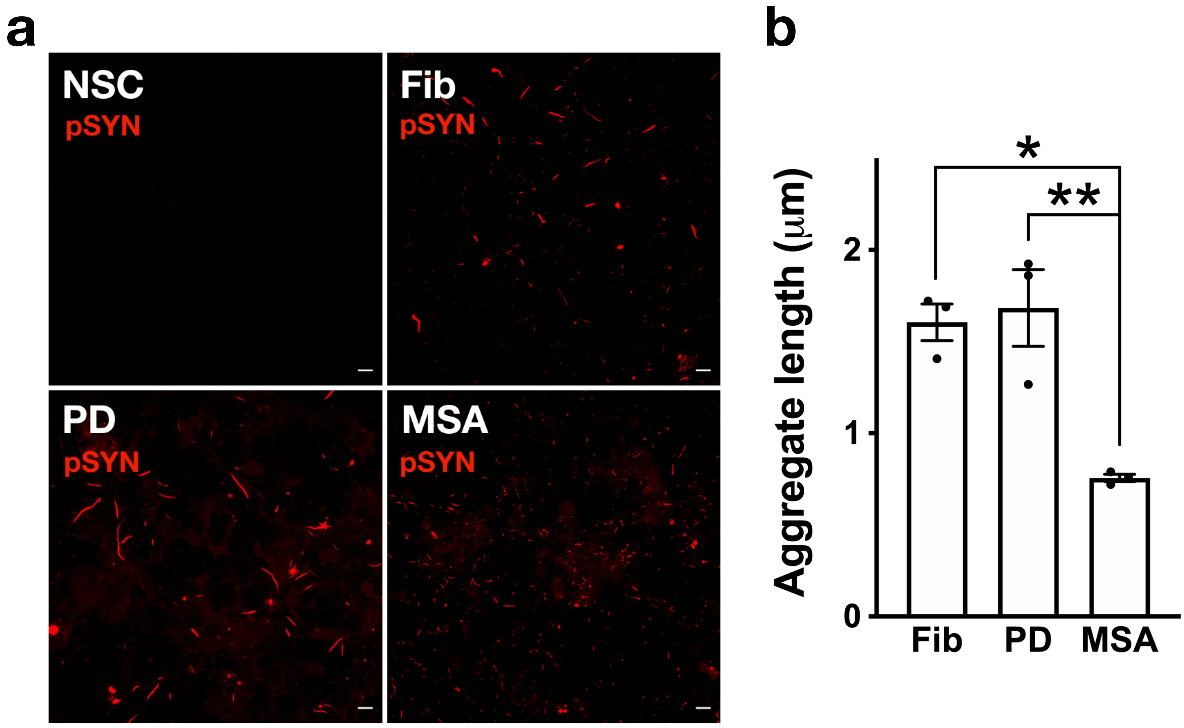

Supplementary Figure 7. Effect of brain-amplified fibrils on healthy control iPSC-derived dopaminergic neurons. a, Confocal images of healthy control dopaminergic neurons treated with *de novo* generated, PD- or MSA-amplified fibrils. Scale bar: 10 μm. Images are representative of three independent differentiations with quantification in panel **b.** As observed with $SNCA^{TRIP}$ neurons, MSA fibrils induced the formation of short pSYN-aggregates, PD fibrils induced the formation of longer aggregates and *de novo* generated fibrils had an intermediate length when analysed with ridge detection (Fiji plugin). In panel **b,** each dot corresponds to one clone per line differentiated once (n=3) and data are mean±s.e.m. **P*=0.0101, ***P*=0.0066 by one-way ANOVA followed by Tukey's multiple comparison test. Source data for **b** is provided as a Source Data file.

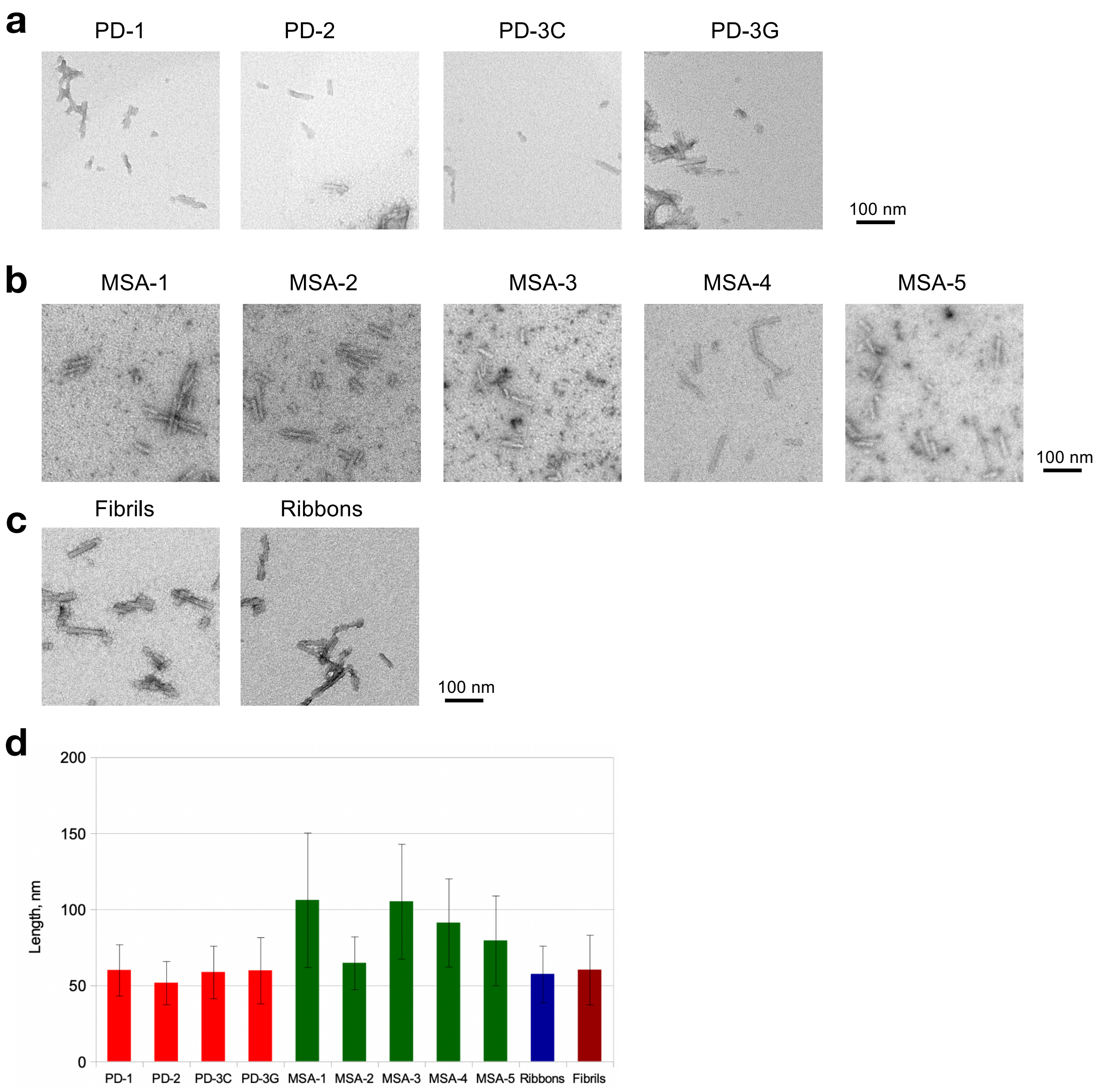

Supplementary Figure 8. Electron microscopy measurements of *de novo* generated fibrils and brain amplified strains after sonication. Assemblies were sonicated under strictly controlled conditions and assessed for filament length. **a**, PD-amplified **b**, MSA-amplified and **c**, *de novo* generated (fibrils and ribbons) strains had overall similar lengths (Scale bar: 100 nm). In panels **a-c**, each TEM image is representative of one independent fibril preparation (n=4 for PD; n=5 for MSA). **d**, The average length of all sonicated fibrils used for seeding was 60 nm. PD-1 (n=102 fibrils), PD-2 (n=115 fibrils), PD-3C (n=100 fibrils), PD-3G(n=92 fibrils), MSA-1(n=115 fibrils), MSA-2(n=105 fibrils), MSA-3(n=102 fibrils), MSA-4(n=110 fibrils), MSA-5(n=102 fibrils), Ribbons(n=104 fibrils), Fibrils(n=116 fibrils). Data are mean±std.dev. Source data for **d** are provided as a Source data file.

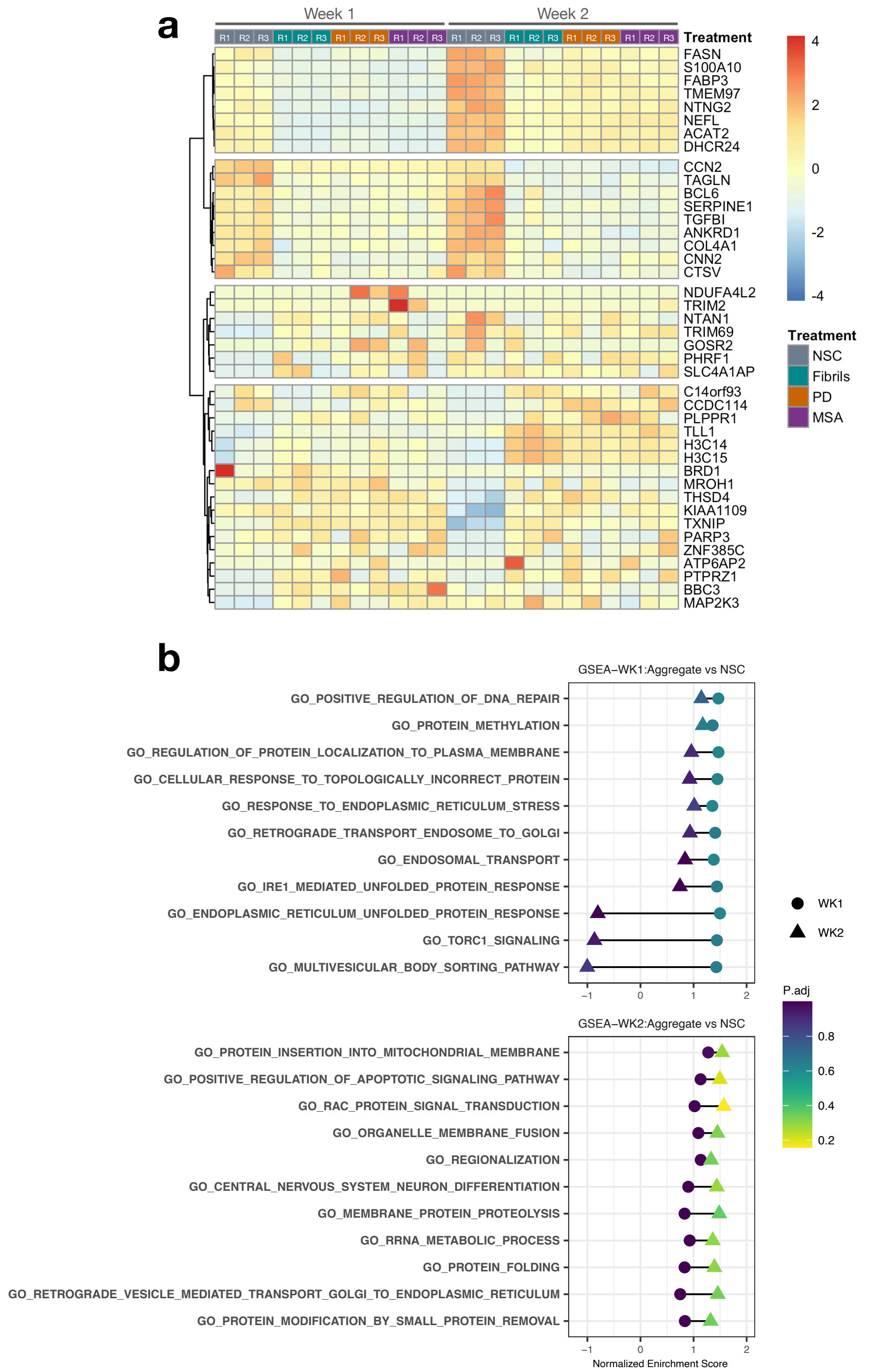

Supplementary Figure 9. Differential gene expression and gene set enrichment analysis in seeded neurons vs non-seeded controls. a, Heatmap of top 15 up and down differentially expressed genes at week 1 and week 2 post seeding, with scaled rlog transformed values across samples for each gene represented by colours on the corresponding row. **b,** Top 20 Gene Ontology (GO) terms identified using Gene Set Enrichment Analysis (GSEA) were enriched in biologically relevant pathways that differed at week 1 from week 2 suggesting a progressive impact of aggregation on organelle homeostasis. Specifically, among the top 20 GO terms, Unfolded Protein Response and endoplasmic reticulum (ER) stress, endosomal and endosome to Golgi transport were up-regulated at week 1 whereas protein folding, membrane protein proteolysis, mitochondrial membrane protein insertion, organelle fusion and apoptosis were detected in week 2. RNASeq data were deposited in GEO under accession number GSE171999.

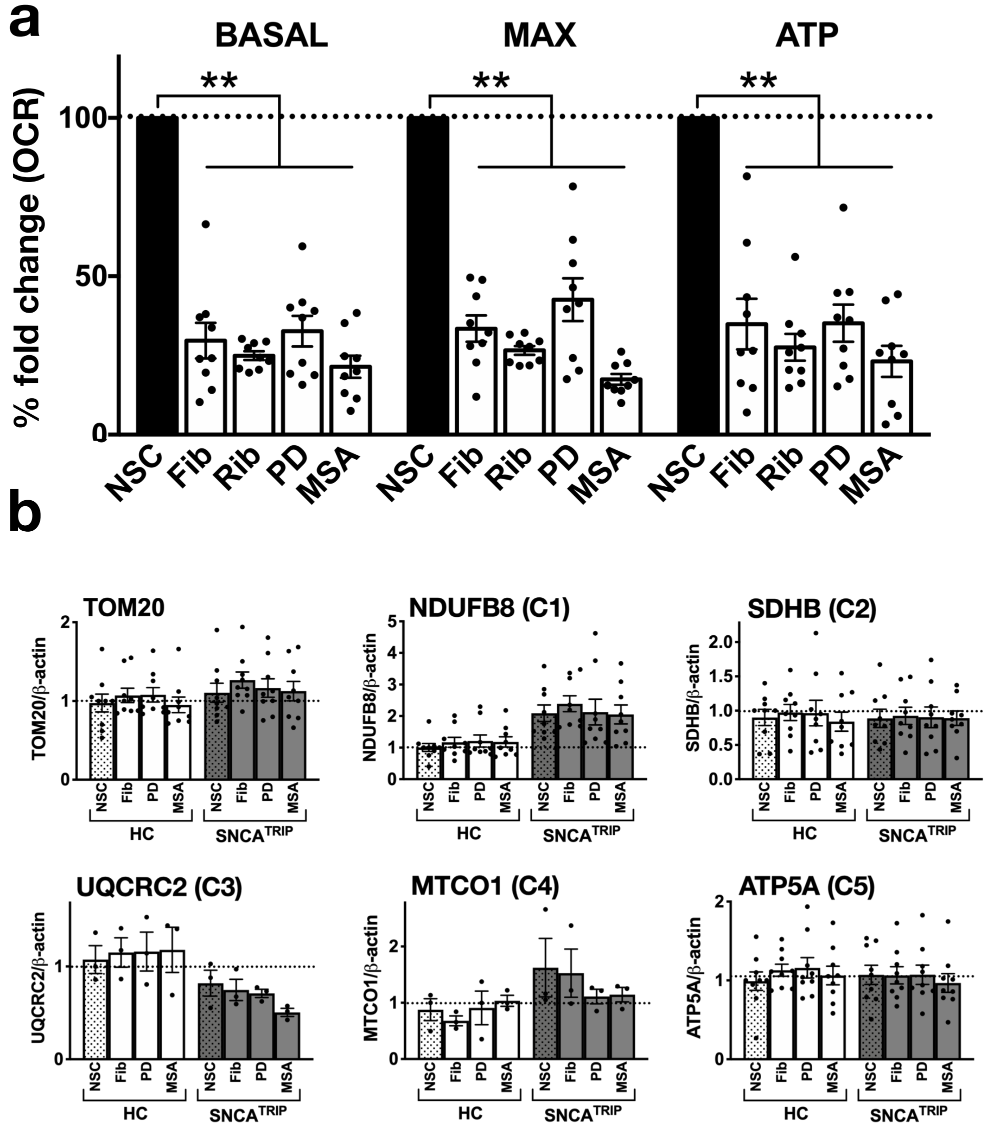

Supplementary Figure 10. Assessment of mitochondrial function and OXPHOS complex proteins in response to treatment with brain-amplified fibrils. a, Healthy control iPSC-derived dopaminergic neurons exhibited reduced OCR, maximal respiration and ATP production 2 weeks post-seeding with *de novo* generated fibrils (1 μM) or brain-amplified fibrils (1 μM). Each dot corresponds to one clone differentiated once from three independent differentiations (n=9). **b,** Immunoblot quantification of TOM20 and respiratory complexes. Each dot corresponds to one clone differentiated once TOM20,n=9; NDUFB8(C1),n=9; SDHB(C2),n=9; UQCRC2(C3),n=3; MTCO1(C4),n=3; ATP5A(C5),n=9. Data are mean±s.e.m. In panel **a**, $^{**}P<0.01$, by one-way ANOVA followed by Tukey's multiple comparison test. Source data for **a** and **b** is provided as a Source Data file.

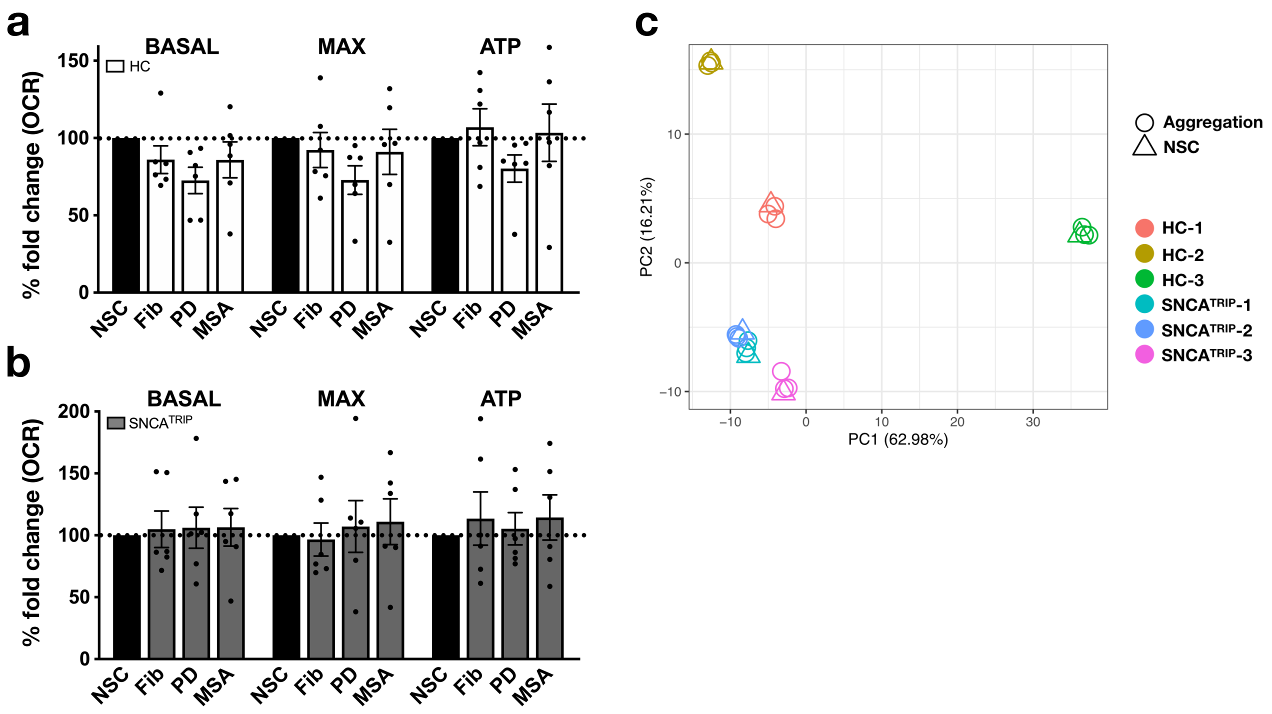

Supplementary Figure 11. Characterisation of aggregation in iPSC-derived dopaminergic neurons treated with 0.1 μM fibrils. Seeded aggregation with either *de novo* generated or brain-amplified fibrils at 0.1 μM did not cause any overt deficit in OCR, maximal respiration and ATP production in **a,** healthy control (*n*=6) or **b,** SNCA^{TRIP} neurons (*n*=6). **c**, Principle component analysis (PCA) on the 1000 most variable protein-coding genes in samples seeded with 0.1 μM seeds (*de novo* fibrils, PD or MSA amplified fibrils) clustered by genomic background rather than aggregate type. For panels **a** and **b** each dot corresponds to one clone differentiated once and data are mean±s.e.m. Statistical significance was calculated by one-way ANOVA followed by Tukey's multiple comparison test. Source data for **a** and **b** is provided as a Source Data file. RNASeq data in panel **c** were deposited in GEO under accession number GSE149632.

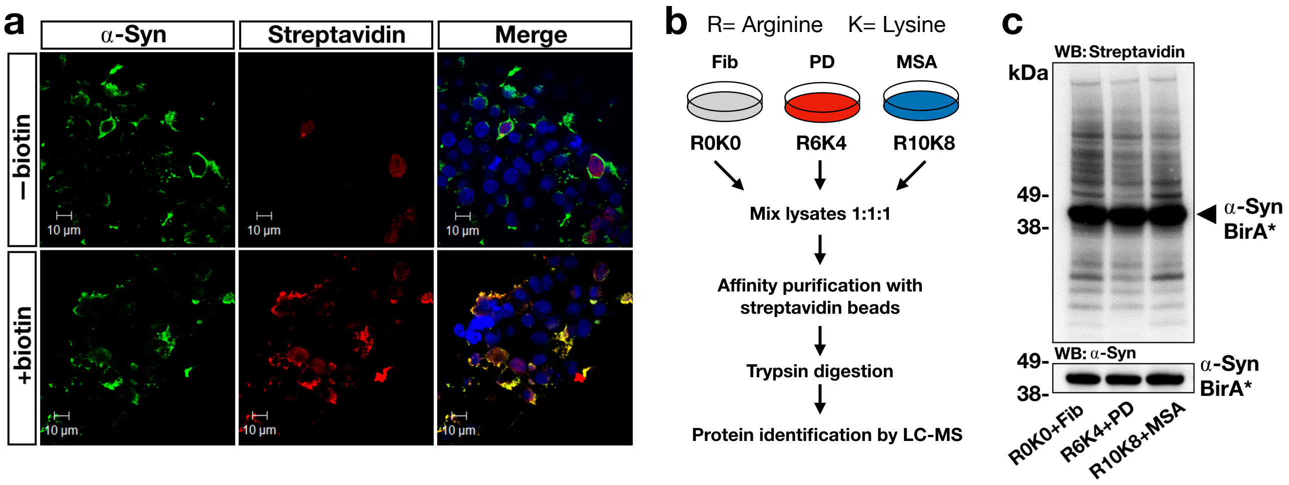

Supplementary Figure 12. Confocal images of cells treated with biotin and biotinylation of SILAC labelled cells, a, Staining pattern of α-Synuclein with streptavidin. Cells expressing α-Syn-BirA* were treated with biotin and stained with α-Synuclein and streptavidin. In the presence of biotin, α-synuclein co-localised with biotinylated proteins. Images are representative of three independent experiments. (Scale bar: 10 μm). **b,** Summary of workflow: SILAC labelled cells were treated with *de novo* generated or brain amplified fibrils and biotin. 48 h post treatment, cells were lysed, run on blot and **c,** probed for streptavidin to test for equal biotinylation. The lysates were pooled together and biotinylated proteins were isolated using streptavidin beads. The bound fraction was then subjected to trypsin digestion and further processed for mass spectrometry. Panel **c** is representative of two independent experiments. R= Arginine, K= Lysine. Source data for **c** is provided as a Source Data file.

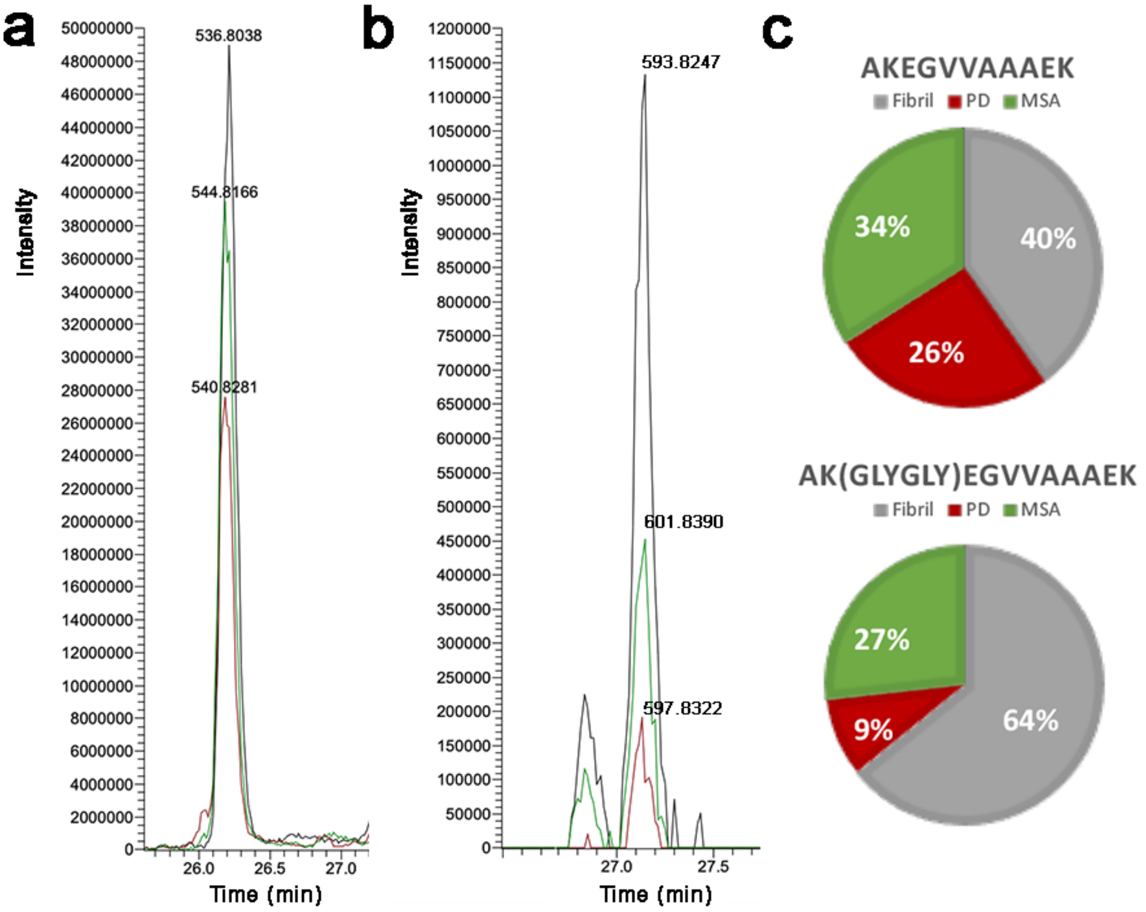

Supplementary Figure 13. SILAC quantitation for K12 containing peptide with and without GlyGly modification. a, Extracted Ion Chromatograms for each monoisotopic SILAC precursor ion showed highest abundance of unmodified K12 peptide in cells seeded with *de novo* generated fibrils (SILAC light @ m/z 536.8), with similar but lower abundance in cells seeded with the PD (SILAC medium @ m/z 540.8) and MSA amplified (SILAC heavy @ m/z 544.8) fibrils. **b,** The corresponding GlyGly modified version of the peptide was mostly detected in cells seeded with *de novo* generated fibrils (SILAC light @ m/z 593.8) and was much less abundant in cells seeded with PD (SILAC medium @ m/z 597.8) or MSA (SILAC light @ m/z 601.8) amplified fibrils. **c**, Relative abundance distribution of mass spectrometry detected signal derived from unmodified K12 peptide (upper) and GlyGly modified K12 peptide (lower). Samples treated with PD and MSA amplified fibrils had much lower amounts of K12 ubiquitinated α-synuclein than samples treated with *de novo* generated fibrils (n=2). The mass spectrometry data were deposited to the ProteomeXchange Consortium via the PRIDE partner repository with the dataset identifier PXD024198.

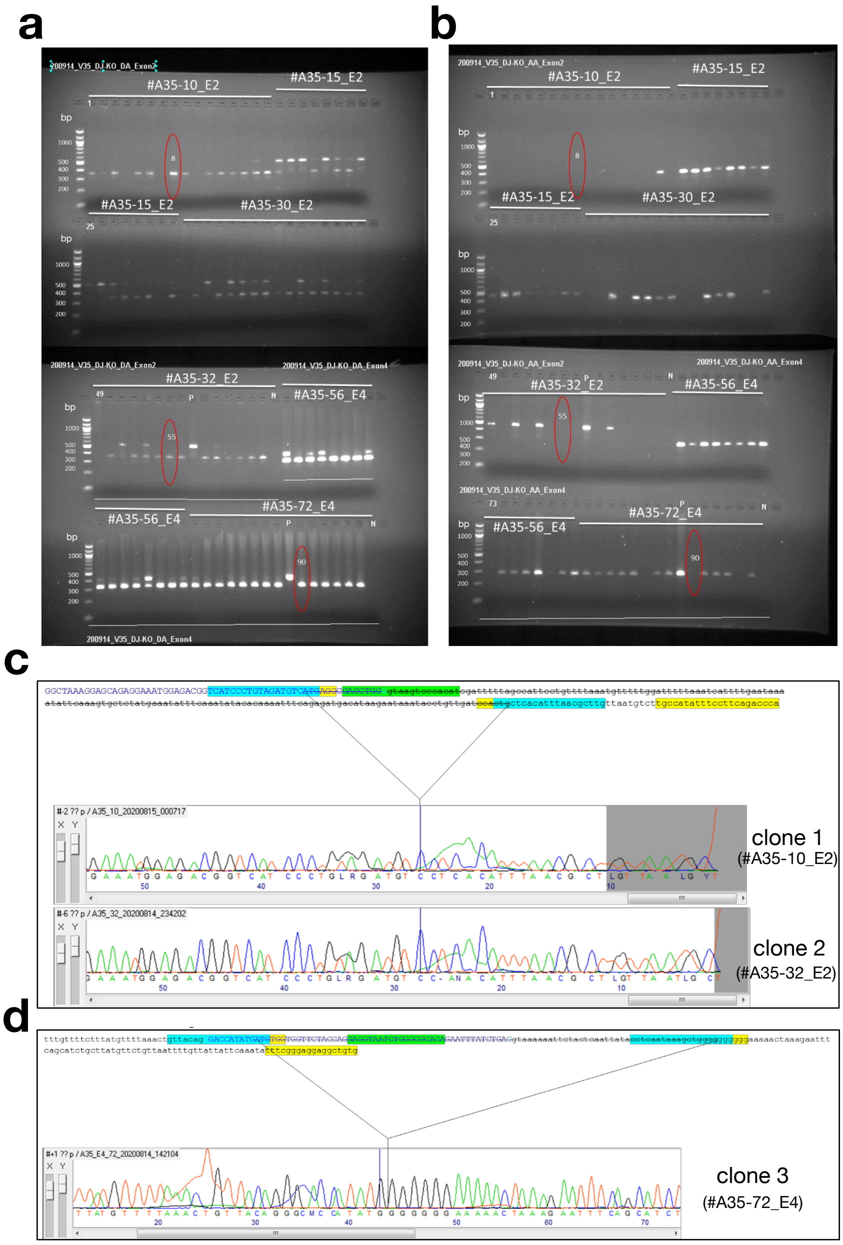

Supplementary Figure 14. Generation of DJ-1 KO clones in SNCA^{TRIP} iPSCs. SNCA^{TRIP} iPSCs were nucleofected with CRISPR complexes formed by two sgRNAs targeting either exon 2 or exon 4 of DJ-1 and the HiFi Cas9 protein. After one week, colonies from each nucleofection were picked and analysed by two primer pairs to identify knockout clones. One primer pair was placed around the deleted region and includes part of the neighbouring intronic region (detection assay; DA) whilst a separate primer pair was placed within the deleted region (absence assay; AA). Positive clones in the DA will have a shorter PCR product, whilst positive clones in the AA will have no product. Selected single cells were expanded and tested by DA and AA PCR for exon 2 and exon 4. **a**, For positive clones in the DA, bands at 362 bp (exon 2) or 341 bp (exon 4) were expected. Unwanted wild-type clones showed higher bands at 531 bp (exon 2) or 431 bp (exon 4). **b**, For positive clones in the AA, no bands were expected. Unwanted wild-type clones showed bands at 464 bp (exon 2) or 284 bp (exon 4). Panels **a** and **b** are representative of one PCR experiment per isolated clone. **c**, Sanger sequencing of SNCA$^{TRIP/DJ-1\ KO}$ clone 1 (#A35-10_E2) and clone 2 (#A35-32_E2) confirmed a deletion of 169 bp at the indicated site. **d**, Sanger sequencing of SNCA$^{TRIP/DJ-1\ KO}$ clone 3 (#A35-72_E4) confirmed a deletion of 90 bp at the indicated site.

	$SNCA^{TRIP/DJ-1KO}$							
	clone 1		clone 2		clone 3		$SNCA^{TRIP}$	
Marker	Allele 1	Allele 2	Allele 1	Allele 2	Allele 1	Allele 2	Allele 1	Allele 2
AMEL	X		X		X		X	
CSF1PO	12		12		12		12	
D13S317	9	11	9	11	9	11	9	11
D16S539	11	12	11	12	11	12	11	12
D18S51	13	22	13	22	13	22	13	22
D19S433	14		14		14		14	
D21S11	30	31,2	30	31,2	30	31,2	30	31.2
D2S1338	21	23	21	23	21	23	21	23
D3S1358	16	17	16	17	16	17	16	17
D5S818	11		11		11		11	
D7S820	9	11	9	11	9	11	9	11
D8S1179	13	14	13	14	13	14	13	14
FGA	20	23	20	23	20	23	20	23
TH01	7	9,3	7	9,3	7	9,3	7	9.3
TPOX	8	11	8	11	8	11	8	11
vWA	15	18	15	18	15	18	15	18

Supplementary Figure 15. Short tandem repeat analysis of CRISPR/Cas9 knockout clones and their corresponding line of origin. The number of allelic repeats tested in each locus matched between all lines, confirming that $SNCA^{TRIP/DJ\text{-}1\ KO}$ clones 1/2/3 were derived from the parent $SNCA^{TRIP}$ iPSC line.

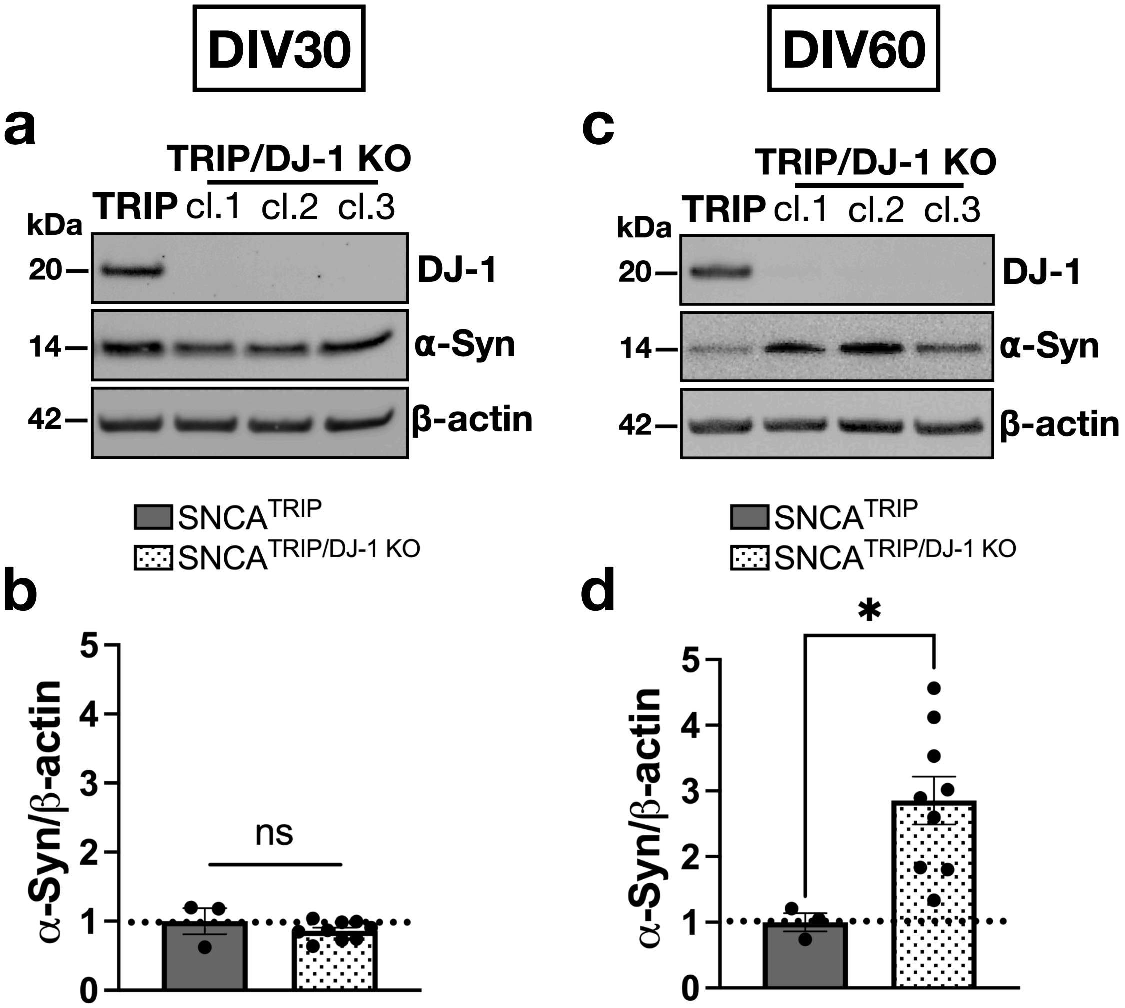

Supplementary Figure 16. Baseline α-synuclein levels increased with time in culture in SNCA$^{TRIP/DJ-1KO}$ compared to SNCA^{TRIP} iPSC-derived neurons. a, Representative immunoblot for neuronal α-synuclein levels at DIV30 showed no difference between parental iPSC-derived SNCA^{TRIP} neurons and iPSC-derived SNCA$^{TRIP/DJ-1\ KO}$ neurons. This was quantified in panel **b** (n=9 for SNCA$^{TRIP/DJ-1\ KO}$ and n=3 for SNCA^{TRIP})**. c**, Representative immunoblot at DIV60 demonstrated increased α-synuclein levels in SNCA$^{TRIP/DJ-1\ KO}$ neurons compared to SNCA^{TRIP}. This was quantified in panel **d** (n=9 for SNCA$^{TRIP/DJ-1\ KO}$ and n=3 for SNCA^{TRIP}). Each dot corresponds to one clone differentiated once and data are mean±s.e.m from 3 independent differentiations. In panel **d**, **P*=0.0178, by two-sided unpaired Student's t-test. Source data for **a, b, c, d** are provided as a Source Data file.
