# Supplementary material for: Phenotypic manifestation of α-synuclein strains derived from Parkinson’s disease and multiple system atrophy in human dopaminergic neurons
Source: Nat Commun. 2021 Jun 21;12:3817. doi: 10.1038/s41467-021-23682-z (PMC8217249; doi:10.1038/s41467-021-23682-z)
Supplement: Supplementary file 3 — Description of Additional Supplementary Files [file 41467_2021_23682_MOESM3_ESM.pdf]

Description of Additional Supplementary Files

Supplementary Data 1: An excel file summarising the results of the proteomic analysis
