# Supplementary material for: Phenotypic manifestation of α-synuclein strains derived from Parkinson’s disease and multiple system atrophy in human dopaminergic neurons
Source: Nat Commun. 2021 Jun 21;12:3817. doi: 10.1038/s41467-021-23682-z (PMC8217249; doi:10.1038/s41467-021-23682-z)
Supplement: Supplementary file 5 — Reporting summary [file 41467_2021_23682_MOESM5_ESM.pdf]

nature research

Corresponding author(s): George Tofaris

Last updated by author(s): Apr 19, 2021

Reporting Summary

Nature Research wishes to improve the reproducibility of the work that we publish. This form provides structure for consistency and transparency in reporting. For further information on Nature Research policies, see Authors & Referees and the Editorial Policy Checklist .

Statistics

For all statistical analyses, confirm that the following items are present in the figure legend, table legend, main text, or Methods section.

n/a	Confirmed	
☐	☒	The exact sample size (*n*) for each experimental group/condition, given as a discrete number and unit of measurement
☐	☒	A statement on whether measurements were taken from distinct samples or whether the same sample was measured repeatedly
☐	☒	The statistical test(s) used AND whether they are one- or two-sided<br>*Only common tests should be described solely by name; describe more complex techniques in the Methods section.*
☐	☒	A description of all covariates tested
☐	☒	A description of any assumptions or corrections, such as tests of normality and adjustment for multiple comparisons
☐	☒	A full description of the statistical parameters including central tendency (e.g. means) or other basic estimates (e.g. regression coefficient) AND variation (e.g. standard deviation) or associated estimates of uncertainty (e.g. confidence intervals)
☐	☒	For null hypothesis testing, the test statistic (e.g. *F*, *t*, *r*) with confidence intervals, effect sizes, degrees of freedom and *P* value noted<br>*Give P values as exact values whenever suitable.*
☒	☐	For Bayesian analysis, information on the choice of priors and Markov chain Monte Carlo settings
☒	☐	For hierarchical and complex designs, identification of the appropriate level for tests and full reporting of outcomes
☒	☐	Estimates of effect sizes (e.g. Cohen's *d*, Pearson's *r*), indicating how they were calculated

Our web collection on statistics for biologists contains articles on many of the points above.

Software and code

Policy information about availability of computer code

Data collection	Confocal microscopy images were obtained using Zeiss LSM 710 c microscope (Zeiss, Germany). Metabolic flux analysis was performed using the Seahorse XFe96 Analyser (Agilent Technologies, USA). TEM in a Jeol 1400 transmission electron microscope was used to assess morphology of the de novo assembled and PMCA amplified αSYN assemblies. Time resolved FRET data were obtained using CLARIOstar Plus (BMG Labtech, Germany). qPCR was performed using Roche LightCycler 480 Instrument (Roche Life Science, Penzberg, Germany). For mass spectrometry samples were analysed on a nLC-MS/MS system consisting of an Orbitrap Fusion Lumos and Dionex Ultimate 3000 (Thermo Fisher). For RNASeq, sequencing was performed with Illumina NovaSeq 6000 (S4) using 150 bp paired end reads.
Data analysis	Statistical analysis was performed using GraphPad Prism version 7 (for macOS). Images and immublots were processed using Image J (v2.1.0/1.53c). Aggregate length was measured using the FIJI ridge detection plugin (v1.4.0). Data analysis for proteomics was performed in Maxquant (v1.6.2.3). Kallisto (v4.0.3) was used for aligning RNAseq data and DESeq2 (v1.30.0) was used for differential gene expression. Cell type composition of bulk RNASeq data was characterised using MuSiC (v0.1.1). Gene Set Enrichment Analysis was performed using Fgsea (v1.16.0). Principal component analysis and plotting was performed in R (v4.0.3). Count data were imported into R using Tximport (v1.18.0).

For manuscripts utilizing custom algorithms or software that are central to the research but not yet described in published literature, software must be made available to editors/reviewers. We strongly encourage code deposition in a community repository (e.g. GitHub). See the Nature Research guidelines for submitting code & software for further information.

Data

Policy information about availability of data

All manuscripts must include a data availability statement. This statement should provide the following information, where applicable:

- Accession codes, unique identifiers, or web links for publicly available datasets
- A list of figures that have associated raw data
- A description of any restrictions on data availability

The data that support the findings of this study have been provided as Source Data. RNASeq data were deposited in GEO as follows: GSE149632 (https://

www.ncbi.nlm.nih.gov/geo/query/acc.cgi?acc=GSE149632) and GSE171999 (https://www.ncbi.nlm.nih.gov/geo/query/acc.cgi?acc=GSE171999). Proteomic data were deposited to the ProteomeXchange Consortium via the PRIDE partner repository with the dataset identifier PXD024198 (http://www.ebi.ac.uk/pride/archive/projects/PXD024198).

Field-specific reporting

Please select the one below that is the best fit for your research. If you are not sure, read the appropriate sections before making your selection.

- [x] Life sciences
- [ ] Behavioural & social sciences
- [ ] Ecological, evolutionary & environmental sciences

For a reference copy of the document with all sections, see nature.com/documents/nr-reporting-summary-flat.pdf

Life sciences study design

All studies must disclose on these points even when the disclosure is negative.

Sample size	For cell line based studies at least three biologically independent experiments were performed to allow statistical analysis. For iPSC we used three different clones and an additional isogenic pair for SNCA Triplication, three healthy control lines and two lines with the A53T mutation as summarised in Suppl. Table 1. Each clone was differentiated 3-5 times to ensure n>3 per condition for statistical analysis.
Data exclusions	No data were excluded from the analysis.
Replication	All experiments were replicated in three or more independent experiments. For Mass spectrometry the samples were biologically replicated twice and consistent changes (e.g. DJ-1) were further validated in at least three independent experiments. For experiments involving the use of iPSC, each datapoint represents a clone differentiated once and at least three independent differentiations were included. Biological replicates (n) are defined as differentiations performed at least one cell-split apart, which is generally at least one week and each clone was differentiated three times. All in vitro studies on the fibril strains were conducted using strain preparations from three Parkinson's brains and five MSA brains. All attempts at replication were successful.
Randomization	Randomization does not apply for cell-based experiments because all available lines were treated in the same fashion and plated in equal numbers for all the experimental conditions.
Blinding	All analyses were performed with the investigators blinded to the experimental conditions. All blinding was performed before data collection and samples were unblinded post-analysis.

Reporting for specific materials, systems and methods

We require information from authors about some types of materials, experimental systems and methods used in many studies. Here, indicate whether each material, system or method listed is relevant to your study. If you are not sure if a list item applies to your research, read the appropriate section before selecting a response.

Materials & experimental systems

n/a	Involved in the study	
[ ]	[x]	Antibodies
[ ]	[x]	Eukaryotic cell lines
[x]	[ ]	Palaeontology
[x]	[ ]	Animals and other organisms
[ ]	[x]	Human research participants
[x]	[ ]	Clinical data

Methods

n/a	Involved in the study	
[x]	[ ]	ChIP-seq
[x]	[ ]	Flow cytometry
[x]	[ ]	MRI-based neuroimaging

Antibodies

Antibodies used	The following primary antibodies were used: The following primary antibodies were used: Anti-PSyn EP1536Y (Abcam #51253; 1:1000 dilution for immunocytochemistry, 1:1000 for fluorescent double labelling, 1:1000 for Western blotting); Anti-alpha-syn Syn-1 (aka Clone42) (BD Biosciences #610787; 1:1000 dilution for Western blotting); Anti-alpha-Synuclein 4B12(Biolegend #807801; dilution1:5000 for Western Blotting); Anti-PSyn antibody 81A (Millipore #MABN826; 1:5000 dilution for filter retardation assay). Anti-alpha-Synuclein 10D2(Merck #MABN633; dilution1:5000 for Western Blotting); Anti-alpha-Synuclein ASynM(clone 4.2 Agrisera #AS132719; dilution1:5000 for Western Blotting); Anti-tyrosine hydroxylase clone LNC1 (Merck Millipore #MAB318; 1:250 dilution for immunocytochemistry); Anti-beta-3 tubulin/TUJ1 (Biolegend #MMS-435P; 1:1500 dilution for immunocytochemistry). Anti-MAP2 (Abcam #5392; 1:2000 dilution for immunocytochemistry). Anti-beta Actin (Abcam #8826; 1:10,000 dilution for Western blotting). Anti-TOM20 (Santa Cruz #FL-145; 1:1000 dilution for Western blotting. Total OXPHOS rodent WB antibody cocktail (Abcam #110413; 1:1000 dilution for Western blotting). Anti-AGE (Advanced Glycated End-products) (Merck Millipore #AB9890; 1:1000 for Western blotting). Anti-PARK7/DJ-1 (Abcam #18257; 1:5000 for Western blotting). Anti-GLO1 (Abcam #129124; 1:5000 for Western blotting). Anti-c-Myc (9E10) (Santa Cruz Biotechnology #sc-40; 10 micrograms per reaction for immunoprecipitation). The following secondary antibodies were used: Alexa Fluor 594-labelled goat anti-rabbit IgG H&L (Abcam #150080; 1:1500 dilution for fluorescent double labelling). Alexa Fluor 488-labelled goat anti-mouse

	IgG H&L (Abcam #150113; 1:1500 dilution for fluorescent double labelling). Alexa Fluor 488-labelled goat anti-chicken IgY H&L (Abcam #150169; 1:1500 dilution for fluorescent double labelling). HRP-labeled goat anti-rabbit IgG H&L (Thermo Fisher #31460; 1:5,000 dilution for Western blotting). HRP-labeled goat anti-mouse IgG H&L (Thermo Fisher #31430; 1:5,000 dilution for Western blotting). HRP-labeled rabbit anti-goat IgG H&L (Thermo Fisher #31402; 1:5,000 dilution for Western blotting). DyLight 680 conjugate anti-mouse IgG H&L (Cell Signalling #5470; 1:10,000 dilution for Western blotting). DyLight 800 PEG conjugate anti-rabbit IgG H&L (Cell Signalling #5151; 1:10,000 dilution for Western blotting).
Validation	Anti-PSyn EP1536Y (Abcam #51253): As per manufacturer's website, EP1536Y reacts with human and mouse pSyn and is suitable for immunofluorescence and immunoblotting. Our results demonstrate that no signal is observed in non-seeded iPSC-derived neurons either by immunoblotting (see Figure 1) or immunofluorescence (see Figure 1). Notably, EP1536Y is one of the few PSyn antibodies that does not cross react with phosphorylated neurofilament (see Rutherford et al., Acta Neuropathologica Communications, 2016).<br>Anti-alpha-synuclein Syn-1 (Clone42, BD Biosciences #610787): Validated on human tissue lysate reproducing appropriate immunoreactive band size and pattern.<br>Anti-alpha-Synuclein 4B12(Biolegend #807801): Validated with recombinant protein and found to recognize residues 103-108 and on human tissue reproducing appropriate immunoreactive band size and pattern.<br>Anti-pSyn antibody 81A (Millipore #MABN826): Validated on human tissue reproducing appropriate immunoreactive pattern.<br>Anti-alpha-Synuclein 10D2(Merck #MABN633): Validated with recombinant protein and found to recognize residues 118-127 and on human tissue reproducing appropriate immunoreactive band size and pattern.<br>Anti-alpha-Synuclein ASynM(clone 4.2 Agrisera #AS132719): Validated with recombinant protein and found to recognize residues 1-15 and on human tissue reproducing appropriate pattern.<br>Anti-tyrosine hydroxylase clone LNC1 (Merck Millipore #MAB318; 1:250): Validated on human tissue and cells reproducing appropriate immunoreactive pattern.<br>Anti-beta-3 tubulin/TUJ1 (Biolegend #MMS-435P): Validated on human tissue and cells reproducing appropriate immunoreactive band size and pattern.<br>Anti-MAP2 (Abcam #5392): Validated on human tissue and cells reproducing appropriate immunoreactive pattern.<br>Anti-beta Actin (Abcam #8826): Validated on human tissue reproducing appropriate immunoreactive pattern.<br>Anti-TOM20 (Santa Cruz #FL-145): Validated on human cells reproducing appropriate immunoreactive band size and pattern.<br>OXPHOS rodent WB antibody cocktail (Abcam #110413): Validated on human tissue reproducing appropriate immunoreactive pattern by Western blotting.<br>Anti-AGE (Merck Millipore #AB9890): Validated on human tissue reproducing appropriate immunoreactive pattern by Western blotting.<br>Anti-PARK7/DJ-1 (Abcam #18257): Validated on human cells reproducing appropriate immunoreactive band size and pattern.<br>Anti-GLO1 (Abcam #129124): Validated on human cells reproducing appropriate immunoreactive band size and pattern.<br>Anti-c-Myc (9E10, Santa Cruz Biotechnology #sc-40): Validated to immunocapture or immunostain c-Myc-tagged fusion proteins expressed in eukaryotic cells.

Eukaryotic cell lines

Policy information about cell lines

Cell line source(s)	HEK293 cells, ATCC®, CRL-1573™<br>All iPSC lines were previously generated and characterised in studies indicated in Suppl. Table 1
Authentication	All iPSC lines were extensively authenticated by confirming their differentiation into dopaminergic neurons with immunofluorescence microscopy and RNASeq analysis as shown in Suppl Figure 1. HEK cell lines and iPSC lines were karyotyped and STR profiled by the supplier. We did not re-authenticate cell lines in-house except for STR profiling of the DJ-1 knockout iPSC lines. For HEK293 cells, fresh stocks from initially derived cells were used for all experiments within 20 passages.
Mycoplasma contamination	All cell lines and iPSC lines were tested and confirmed negative for mycoplasma contamination during the course of the experiments.
Commonly misidentified lines<br>(See ICLAC register)	No commonly misidentified cell lines were used in this study

Human research participants

Policy information about studies involving human research participants

Population characteristics	Fresh frozen tissue was dissected from 3 sporadic PD, 5 MSA and 3 control brains. Anterior cingulate cotex was dissected from each PD and control brain, whereas cerebellum was dissected from each MSA and control brain. For one PD case we also used frozen tissue from the temporal gyrus. All PD and MSA cases exhibited alpha-synuclein positive inclusions on neuropathological examination.
Recruitment	The PD and control brain samples were obtained from the Oxford Brain Bank and the MSA brain samples were obtained from the Parkinson's UK Brain Bank. Consent forms were signed by the subjects themselves or their next of kin in accordance with UK ethics laws.
Ethics oversight	Ethical approval for the use of human tissue was obtained from the Oxford C REC Ethics Committee- No15/SC/0639

Note that full information on the approval of the study protocol must also be provided in the manuscript.
